# Self-Organization of Blood Pressure Regulation: Clinical Evidence

**DOI:** 10.3389/fphys.2016.00113

**Published:** 2016-03-30

**Authors:** Jacques-Olivier Fortrat, Claude Gharib

**Affiliations:** ^1^UMR Centre National de la Recherche Scientifique 6214 Institut National de la Santé et de la Recherche Médicale 1083 (Biologie Neurovasculaire et Mitochondriale Intégrée), Faculté de Médecine d'AngersAngers, France; ^2^Université Claude Bernard Lyon 1, Faculté de Médecine Lyon EstLyon, France; ^3^Centre International d'OstéopathieSaint Etienne, France

**Keywords:** autonomic nervous system, baroreflex, blood pressure control, non-linear dynamics, self-organized criticality, syncope

## Abstract

The pathogenesis of vasovagal syncope has remained elusive despite many efforts to identify an underlying dysfunction. Catastrophe theory explains the spontaneous occurrence of sudden events in some mathematically complex systems known as self-organized systems poised at criticality. These systems universally exhibit a power law initially described in earthquake occurrence: the Gutenberg Richter law. The magnitude plotted against the total number of earthquakes of at least this magnitude draw a straight line on log-log graph. We hypothesized that vasovagal syncope is a catastrophe occurring spontaneously in the cardiovascular system. We counted the number and magnitude (number of beats) of vasovagal reactions (simultaneous decreases in both blood pressure and heart rate on consecutive beats) in 24 patients with vasovagal symptoms during a head-up tilt test and 24 paired patients with no symptoms during the test. For each patient, we checked whether vasovagal reaction occurrence followed the Gutenberg Richter law. The occurrence followed the Gutenberg Richter law in 43 patients (correlation coefficient |*r*| = 0.986 ± 0.001, mean ± SEM) out of 48, with no difference between patients with and without symptoms. We demonstrated that vasovagal syncope matches a catastrophe model occurring in a self-organized cardiovascular complex system poised at criticality. This is a new vision of cardiovascular regulation and its related disorders.

## Introduction

Vasovagal reaction is a transient failure in cardiovascular regulation, leading to cerebral hypoperfusion, and eventually to syncope (Grubb, 2005; da Silva, 2014). Some vasovagal syncopes are obviously triggered (blood/injury phobia and strong emotion, Accurso et al., 2001). However, most of them occur in the standing position, but are not directly triggered by position change since firstly they occur after prolonged standing (mean of 17 min on a tilt table); secondly they occur occasionally while the patient is standing, with no symptoms most of the time (Fitzpatrick et al., 1991; Grubb, 2005). Vasovagal reaction should not be confused with orthostatic hypotension (da Silva, 2014; Raj, 2014). The main hypothesis to explain vasovagal syncope is that the standing decrease in venous return leads to an “empty heart” with increased inotropy. The resulting hypercontractile state activates heart mechanoreceptors, resulting in bradycardia and hypotension (Grubb, 2005; da Silva, 2014; vasovagal reaction).

Several authors suggest an underlying (potentially unknown) disorder and attempted to identify a dysfunction or genetic cause (Marrone et al., 1999; Béchir et al., 2003; Iwase et al., 2014; Komiyama et al., 2015). However, occasional and transient failure of a regulatory mechanism in an apparently healthy cardiovascular system is difficult to explain and the exact mechanism of vasovagal syncope remains elusive (Mosqueda-Garcia et al., 2000; Grubb, 2005).

Vasovagal syncope is a very common phenomenon (40% of the population) that could happen to anyone including young, healthy people (da Silva, 2014; Raj, 2014). Vasovagal syncope is so frequent that we suggest there is no underlying disorder. Vasovagal syncope is rather a spontaneous event that could occur in any normal cardiovascular system. Linear systems are not supposed to present such spontaneous occasional and transient dysfunctions without a trigger, but the cardiovascular system exhibits some non-linear patterns like chaos and fractal scaling (Kobayashi and Musha, 1982; Marrone et al., 1999; Castiglioni et al., 2011; Porta et al., 2015).

Spontaneous catastrophes occur in some kinds of non-linear complex systems: self-organized systems poised at criticality (Chen et al., 1991). Self-organization explains earthquakes in plate tectonic. Suggesting self-organization poised at criticality for the cardiovascular system is not all that provocative since this principle has been suggested as a general law for biological (including the cardiovascular system) as well as physical systems (Bak et al., 1987; Bak, 1996; Mora and Bialek, 2011; Struzik, 2014). Studying all events and not only “big ones” has remarkably improved understanding about earthquakes. Their magnitude is inversely proportional to their number and produces a straight line on a logarithmic graph according to the Gutenberg-Richter law (earthquake magnitude scale is named after Richter). Observing this law demonstrates that the system is self-organized (Chen et al., 1991). We hypothesized that the cardiovascular system is self-organized and checked whether the distribution of vasovagal reactions follows the Gutenberg-Richter law.

## Materials and methods

### Ethical approval

Patients received a complete description of the experimental procedure before giving their written informed consent. The Comité de Protection des Personnes d'Angers (Angers Committee for the Protection of Persons), France approved the experiment which is in accordance with the declaration of Helsinki, Finland.

### Patients

We proposed the experiment to patients who came to our department for advice or evaluation of their (near) transient loss of consciousness. One hundred consecutive patients who gave their informed consent were included. All of these patients benefited from a syncope evaluation that included at least their medical history, a clinical examination, electrocardiogram, and a 45 min head-up tilt test [or less in the event of (pre)syncopal symptoms, see procedure below]. Of these 100 patients, 70 patients had a normal electrocardiogram, no history of heart disease, and no previously diagnosed orthostatic hypotension. The head-up tilt test identified three patients with orthostatic hypotension and five patients with postural orthostatic tachycardia syndrome out of these 70 patients. Of the remaining 62 patients, 34 patients had (pre)syncopal Vasovagal Symptoms during the head-up tilt test. We split the patients into two groups according to whether or not they suffered (pre)syncopal Vasovagal Symptoms during the head-up tilt test (VS+ and VS−, respectively) and matched them for age and sex. Finally, 24 patients with (pre)syncopal Vasovagal Symptoms were matched with patients with no symptoms during the head-up tilt test.

### Head-up tilt test

We monitored 12-lead electrocardiogram and blood pressure (MAC vu, Marquette, Milwaukee, WI, USA; and Finometer, FMS system, Amsterdam, Netherlands, with sampling frequency set at 500 Hz, AT-MIO-16, 12 bits, Labview 5.1, National Instruments, Austin, TX, USA) of the patient lying on a table (Akron A8622, Electro-Medical Equipment, Marietta, GA, USA). We began to record these cardiovascular signals after a period of at least 10 min of adaptation in a horizontal (supine) position. Recording began with 10 min in this position and continued while the table was tilted over 20 s to the head-up position (inclination of 70°) that was maintained for 45 min [or less in the event of (pre)syncopal Vasovagal Symptoms].

### Signal analysis

We determined RR-interval on ECG signals off-line by means of a peak detection algorithm. We visually inspected all the ECG signals to identify R peak misdetections and ectopic beats. We manually deleted false detections. We observed ectopic beats in only seven patients (three VS+ and three VS−); they were sparse (maximum of two per 5 min on two VS− patients). We determined the true mean blood pressure on a beat-by-beat basis (mean of instantaneous blood pressure during each heart beat). We determined RR-intervals and blood pressure at the resolution of the data acquisition.

**Figure 1:**
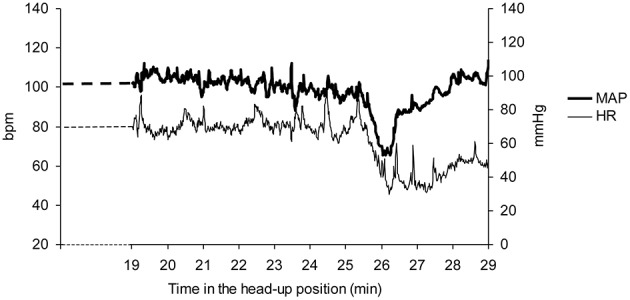
**A vasovagal syncope during a head-up tilt test in a patient**. Note the drop in mean arterial blood pressure (MAP) occurring after about 25 min in the head-up position and the accompanying drop in heart rate (HR). The end of the head-up position is marked by the recovery of blood pressure. Bpm: beats per minute.

**Figure 2:**
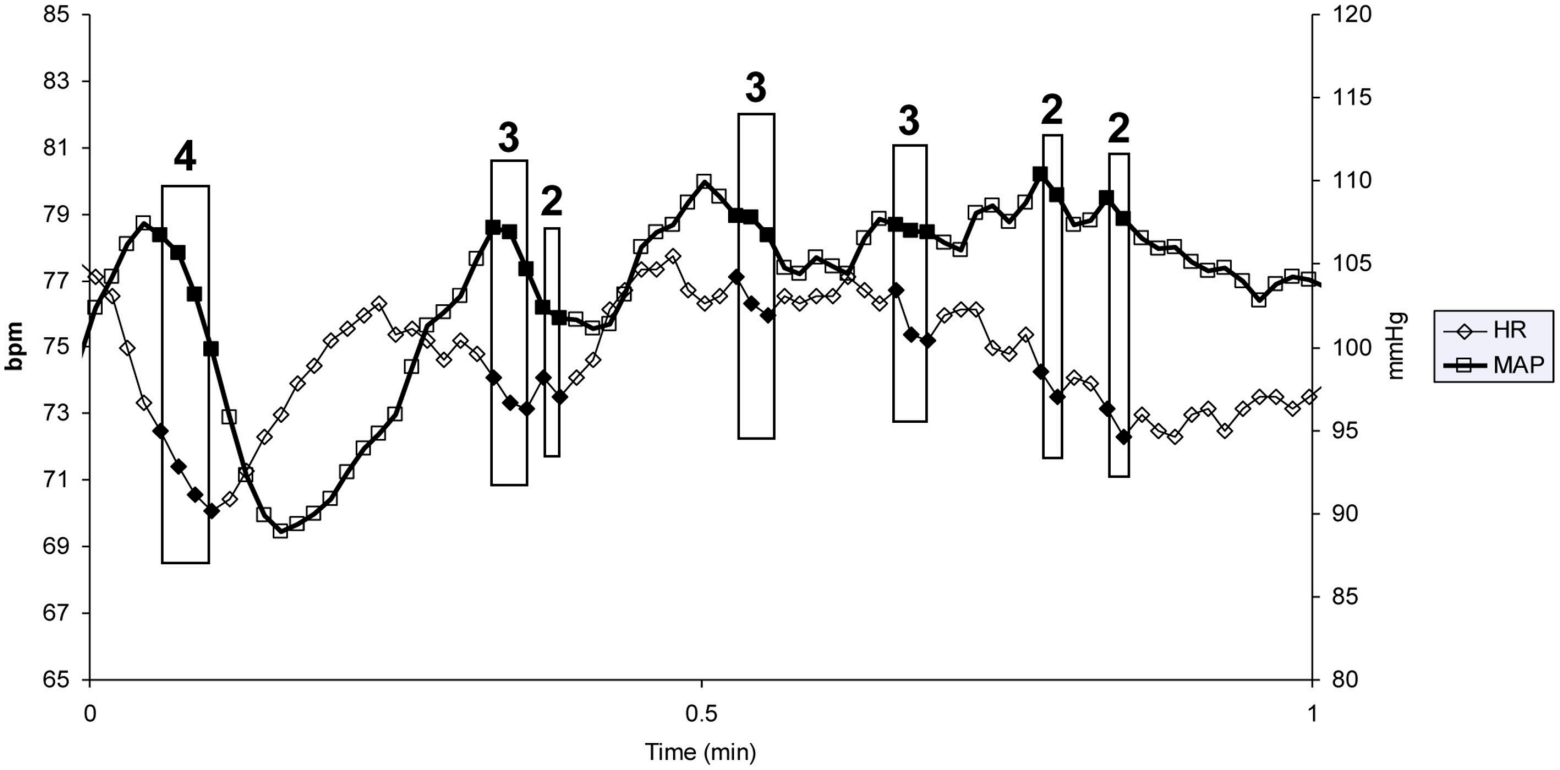
**One minute of a patient's heart rate (HR) and mean blood pressure (MAP) monitoring**. Each heart beat is indicated by a diamond (HR) and a square (MAP). Each box indicates a vasovagal event. The magnitude of the event (in number of beats) is given above the box (bpm: beats per minute).

Vasovagal syncope is characterized by a drop in blood pressure not compensated by baroreflex mediated tachycardia, but surprisingly accompanied by bradycardia until syncope occurred (Figure 1). We studied all the falls in blood pressure accompanied by bradycardia, not only “big ones” leading to (pre)syncope. We counted the number of times that blood pressure and heart rate simultaneously fell on consecutive beats and the number of beats during such events (number and magnitude of vasovagal events, respectively; Figure 2) during the 55 min monitoring of these patients (10 min supine and 45 min in head-up position or less in the event of symptoms). We did not consider any lag between mean arterial pressure and RR-interval for two main reasons. Firstly, definition of vasovagal reaction has not included such lags (Grubb, 2005; da Silva, 2014). Secondly, there is no consensus about such lags when assessing the beat-by-beat cardiovascular regulation like baroreflex estimation by means of sequence methods (Laude et al., 2004).

For each patient, we plotted magnitude against the number of vasovagal events on a decimal logarithmic graph.

### Statistics

We performed statistics using SPSS software (SPSS 15.0, Chicago, IL, USA). We plotted the number of vasovagal events according to their magnitude on a log-log plot and checked whether the distribution of values followed a straight line, by means of linear regression. We considered that the distribution fitted a straight line when |*r*| > 0.95. Such a high cut-off is usual in exact sciences in which most of the Gutenberg Richter laws have been described (Bak, 1996).

We compared matched patients with and without (pre)syncopal Vasovagal Symptoms during the 45 min head-up tilt test (VS+ and VS−) by means of a paired T test.

We set the statistical significance at *p* < 0.05.

## Results

VS+ and VS− patients had comparable anthropomorphic characteristics, medical history, heart rate, blood pressure, and treatments (Table 1).

**Table 1:** **Patient characteristics**.

**Characteristics**	**VS+**	**VS−**
Number of patients (n)	24	24
Female (n)	16	16
Age (years)	39 ± 3	39 ± 3
Weight (kg)	67 ± 2	69 ± 3
Height (m)	1.66 ± 0.01	1.69 ± 0.02
Heart rate (bpm)	67.3 ± 1.6	65.9 ± 1.4
Systolic blood pressure (mmHg)	127.4 ± 4.0	130.8 ± 3.7
Diastolic blood pressure (mmHg)	74.0 ± 3.1	76.5 ± 4.2
HUTT (min)	22 ± 2	45 ± 0
Cardiovascular treatment (n)	4	2
Non cardiovascular treatment (n)	9	8

*VS+, patients with Vasovagal Symptoms during the head-up tilt test; VS−: patients without Vasovagal Symptoms during the head-up tilt test. HUTT: duration of the head-up tilt test. Number (n) or mean ± SEM is included, bpm: beats per minute. Two patients were on low doses of β-blockers for their vasovagal syncope but stopped taking them for 48 h before the test; other cardiovascular treatments were different kinds of antihypertensive drugs and statins*.

**Figure 3:**
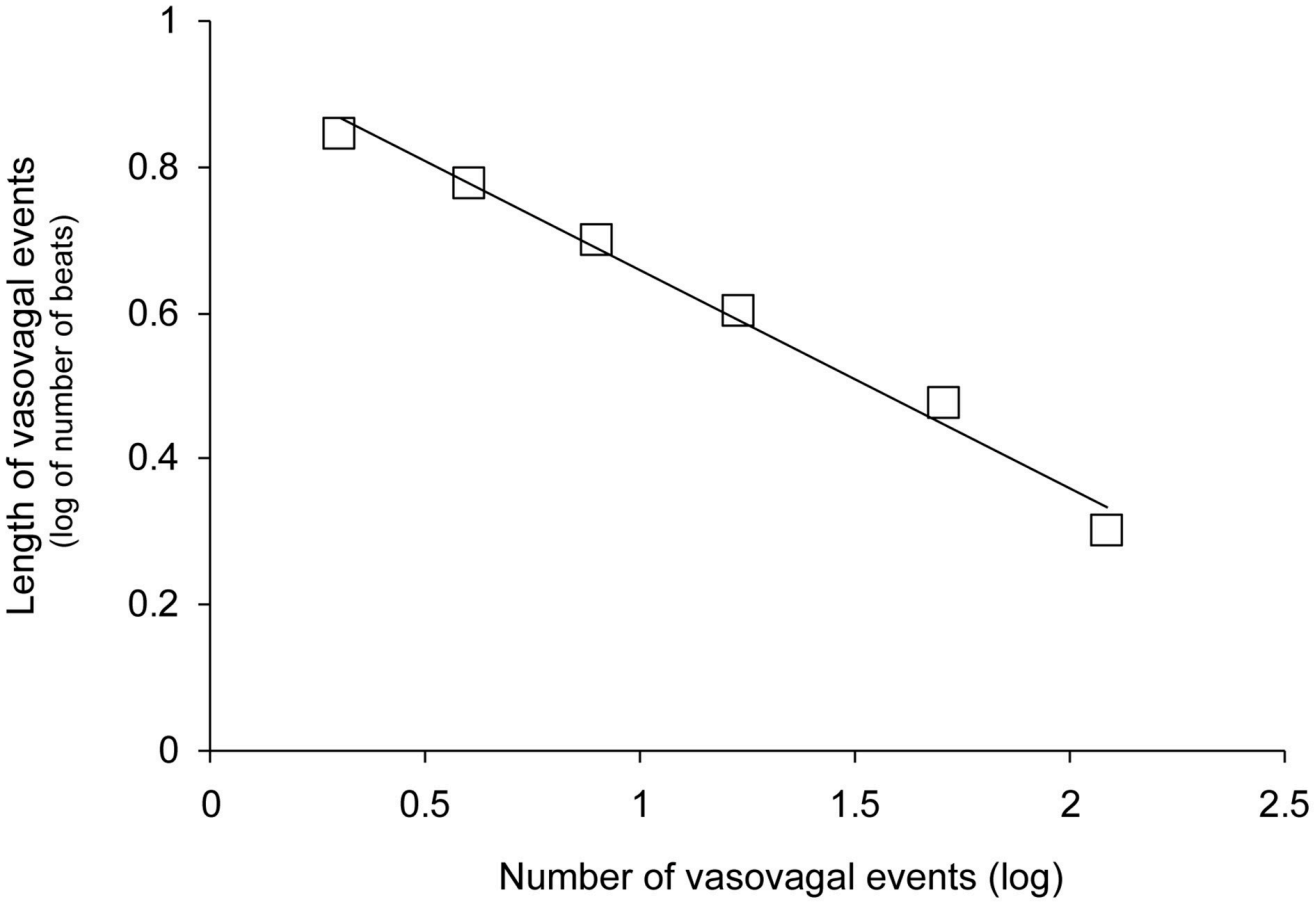
**Distribution of vasovagal events for one patient according to event magnitude (length in number of beats) on a decimal logarithmic graph**. This distribution is a straight line (*y* = −0.30x + 0.96 for this patient without vasovagal symptoms and |*r*| = 0.993) according to the Gutenberg-Richter law. This law is a main feature of self-organized complex systems poised at criticality.

We counted 5.9 ± 0.7 vasovagal events per minute (data is presented with their mean ± SEM). Their maximal magnitude (or maximal length) was 5.1 ± 0.2 beats with a maximum of eight beats. High-magnitude vasovagal events were too scarce in four VS+ patients to perform the analysis because of short monitoring time owing to positive outcome (only two points on the logarithmic graph with |*r*| = 1). We observed a Gutenberg-Richter distribution of these vasovagal events in 43 patients of the 44 remaining patients (24 VS− and 19 VS+) with a high correlation coefficient (|*r*| = 0.986 ± 0.001, Figure 3). In one patient (VS+) the correlation coefficient was not high enough to confirm a straight-line distribution (|*r*| = 0.934). We did not observe any difference between patients with a positive and a negative outcome (Table 2).

**Table 2:** **Group comparison**.

	**VS+**	**VS−**
Number of events/min	6.83 ± 1.39	5.15 ± 0.58
Magnitude (number of beats)	4.95 ± 0.27	5.09 ± 0.25
|*r*|	0.967 ± 0.007	0.972 ± 0.004

*VS+: patients with Vasovagal Symptoms during the head-up tilt test, VS−: patients without Vasovagal Symptoms during the head-up tilt test. No significant differences*.

## Discussion

The present study showed that the vasovagal events are spread according to the Gutenberg-Richter law. This finding strongly supports the hypothesis that the cardiovascular system is self-organized, and explains vasovagal syncope.

### Power laws are common in nature

A straight line drawn in logarithmic graph is commonly observed and is called power law (see Bak 1996). Most of these power laws are incidental findings and their meaning has remained unclear. Gutenberg and Richter also incidentally noted such a power law for earthquake occurrence. However, Bak (1996) demonstrated that Gutenberg Richter law of earthquake occurrence is the result of the self-organized poised at criticality nature of tectonic plate dynamics. Power laws are also common in biology known as Kleiber rules or scaling laws (West et al., 1997). Scaling laws are also observed in the cardiovascular system like the one that links heart rate and body weight across species (West et al., 1997).

### Self-organization in biological systems

Cardiovascular regulation is usually viewed through the homeostasis theory that implies a regulatory loop, negative feedback, and a reference value (the set point). In contrast, self-organization implies numerous sub-units and interaction between opposing mechanisms. Self-organized systems do not have a leader or central controller, and set point. In biology, self-organization was first demonstrated in social animals such as shoals of fish or flocks of birds (Camazine et al., 2001). The sub-units are the individuals, and opposing mechanisms are the simple rules that drive their behavior (if one of my neighbors comes closer, I move away; and if he moves away, I move closer). The functioning of opposing mechanisms leads to a sense of behavior driven by a leader (or a central controller) or to a sense of a set point and reference value.

### Self-organization of physiological functions

It is difficult to imagine how the behavior of a shoal of fish could apply to a physiological function and to the cardiovascular system of a single organism. However, Schöner and Kelso (1988) elegantly demonstrated in a seminal publication that control of movement is self-organized. They proposed that sub-units are the several processes needed for accurate movements that include decision making, learning, sensory perception, and the stretch reflex. Opposing mechanisms are negative and positive feedbacks that interact. The main advantage of self-organization is that it provides an efficient way of coordinating these different kinds of processes and controlling a whole chain of muscles and joints (Kelso, 1995). Homeostatic organization with central integration and negative feedbacks would lead to cumulative delays that are not in accordance with the fast and accurate movements needed to cope with daily life activity and survival (Schöner and Kelso, 1988; Kelso, 1995). Blood pressure control involves many systemic, regional, and local regulations (Guyton et al., 1972 gave a picture of the complexity of blood pressure control) that would be the sub-units. Moreover, fast and accurate blood pressure control is also critical for survival. Self-organization would be an efficient way of coordinating all these regulatory mechanisms.

### The normal cardiovascular function of patients with vasovagal syncope

The lack of difference between the two groups of patients is in accordance with the unremarkable clinical examination of patients with vasovagal syncope who do not stand out from patients with no symptoms. (Grubb, 2005; da Silva, 2014; Raj, 2014). Moreover, this lack of difference is also in accordance with the hypothesis that vasovagal syncope may occur in any normal cardiovascular system with no underlying disorder. Some studies claim for a difference between patients with vasovagal symptoms and patients with no symptoms (see as example: Marrone et al., 1999; Béchir et al., 2003; Iwase et al., 2014; Komiyama et al., 2015). However, these findings have not usually been repeated and could not be included in reviews nor in expert consensus (Brignole et al., 2004; Grubb, 2005; da Silva, 2014; Raj, 2014). Finally, the lack of difference between the two groups of patients also supports our hypothesis that vasovagal syncope is a catastrophe spontaneously occurring in a normal self-organized cardiovascular system poised at criticality.

### Vasovagal events

To the best of our knowledge, we are the first to suggest that any events during which blood pressure and heart rate are both decreasing, are vasovagal reactions including times when they do not lead to symptoms. We suggest that such events without symptoms are “low magnitude” vasovagal reactions. However, we are not the first to note that such events are common, including in healthy subjects (Hughson et al., 1993; Legramante et al., 1999, 2001). Legramante et al. (2001) suggested that they result from a positive feedback mechanism, confirming a previous observation by Pagani et al. (1982). Thus, cardiovascular function may involve well-known negative feedbacks (such as the baroreflex) but also the more recently described positive ones. Cardiovascular function might result from interactions between these negative and positive feedbacks as with control of movement and any self-organized system. It is also important to note that these events are not the same as these used to determine the baroreflex sensitivity by means of sequence methods (Di Rienzo et al., 2001; Laude et al., 2004). Actually, baroreflex sequences are the opposite of vasovagal events since the formers are defined by a change in heart rate accompanied by change in blood pressure in the opposite direction (Laude et al., 2004).

### Limitations of the homeostatic model

The homeostasis view faces an unsolved conundrum. Homeostasis needs a set point (a reference value cast in stone) but, paradoxically, it also needs to constantly tune this set point according to continuous changes in environment and demand. This set point tuning is called “resetting.” However, integrating cardiovascular regulation resetting into the homeostatic model is full of complexities (Koushanpour, 1991; Schwartz and Stewart, 2012). Self-organized systems miss the set point and, by nature, they are flexible and spontaneously adapt to changes in environment and demand. Resetting cardiovascular regulation to high blood pressure values is one of the main hypotheses to explain hypertension. Self-organization solves the resetting conundrum, ruling out the resetting hypothesis for hypertension. Interestingly, self-organization also explains the 1/f pattern of heart rate variability spectral analysis: This pattern is a main characteristic of the dynamics of self-organized systems (Kobayashi and Musha, 1982; Bak et al., 1987).

### Study limitations

The main limitation of our study is that the range of vasovagal reaction magnitudes was small: about two units, compared with about 9 units in the case of earthquakes. However, the seminal review of self-organized criticality mentions several dynamic systems with comparable small-magnitude range such coastal fractality, sediment deposition, pulsar glitches, game of life, and the punctuated equilibrium model of Evolution (Bak, 1996; see also Supplementary Material).

Our hypothesis did not imply looking for any correlation between number and magnitude of vasovagal events but only checking for the goodness of linear fit, i.e., whether a power law is present. Moreover, the design of our study did not allow for looking for such a correlation because of the too short duration of data recordings. They are self-limited in case of vasovagal symptom occurrence and they are limited by the challenge of too long duration of standing position in other cases.

### Conclusion

Our study demonstrated that vasovagal syncope matches a catastrophe model occurring in a self-organized cardiovascular complex system poised at criticality. Diseases not caused by external microbial or toxic agents are usually due to the failure of a physiological system. Here we demonstrated that a normal functioning cardiovascular system entails vasovagal syncope. This is a new vision of the cardiovascular system that provides new insights into blood pressure regulation and its related disorders.

## Author contributions

JOF: conception, design, acquisition, analysis, and drafting of the work; JOF, CG: interpretation of data, revising the work critically for important intellectual content, and final approval of the version to be published.

## Funding

This study was supported by the Centre National d'Etudes Spatiales (CNES, grant # 2014/4800000763). The funder had no role in study design, data collection and analysis, decision to publish, or preparation of the manuscript.

### Conflict of interest statement

The authors declare that the research was conducted in the absence of any commercial or financial relationships that could be construed as a potential conflict of interest.
