# Supplementary material for: Self-Organization of Blood Pressure Regulation: Clinical Evidence
Source: Front Physiol. 2016 Mar 30;7:113. doi: 10.3389/fphys.2016.00113 (PMC4812062; doi:10.3389/fphys.2016.00113)
Supplement: Supplementary file 1 [file Presentation1.PDF]

Supplementary Material

Self-organization of blood pressure regulation:

Clinical evidence

Jacques-Olivier Fortrat1*, Claude Gharib2

*** Correspondence:** Jacques-Olivier FORTRAT, jofortrat@chu-angers.fr

1 Supplementary Data

1.1 Surrogate data

To confirm the linear relationship observed in logarithmic graphs, we performed the same analysis on a surrogate series. A surrogate series was obtained by randomizing the chronological order of RR-interval and blood pressure observed in a subject. We applied the same analysis to the 48 surrogate series as to the actual 48 described in the Methods section. We performed the linear regression once more on the actual series, but on the same range of magnitude as their matching surrogate series for comparison between surrogate and actual series. We compared actual and surrogate series by means of a paired T test.

Randomization decreased the number of vasovagal events (1.7 ± 0.1 events per minute in the surrogate series, $p < 0.001$). The maximum length of these events was also shorter in the surrogate series compared with the actual ones (3.0 ± 0.0 beats in the surrogate series, $p < 0.001$). The slope of linear regression was significantly different between the surrogate and actual series (Supplementary Figure 1). The surrogate series clearly demonstrated that vasovagal events are not random and testify to an underlying physiological mechanism. They also clearly demonstrated that the linear relationship shown in the actual series is not random.

2 Supplementary Figure

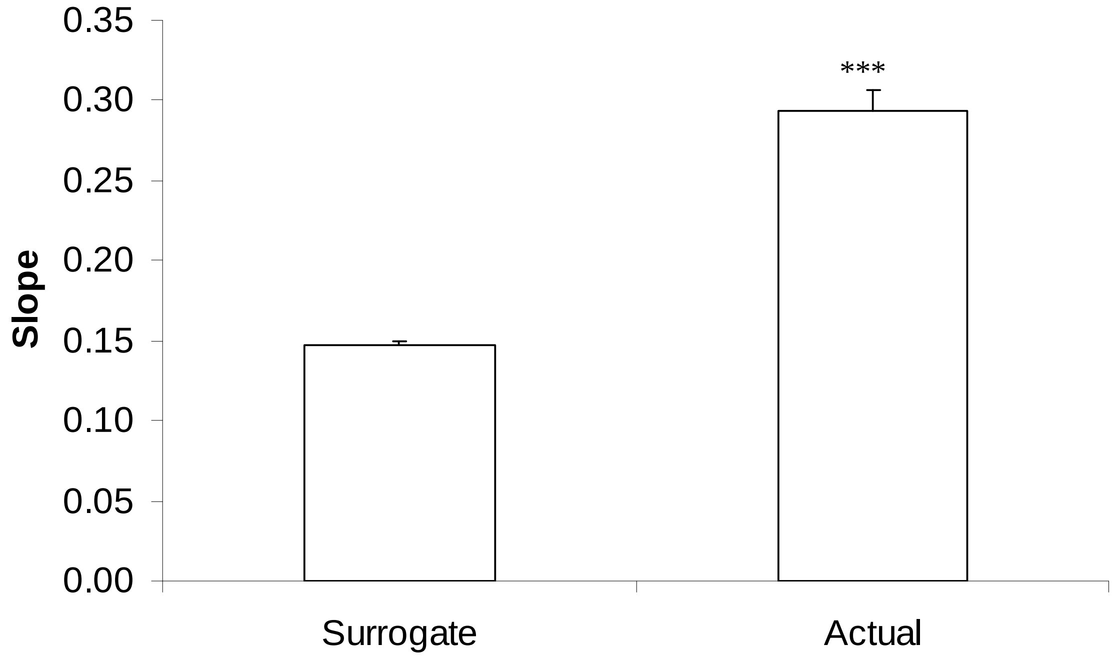

Supplementary Figure 1. Slope of the distribution of vasovagal events (see Figure 2) in the surrogate random series and the actual series. *** $p \leq 0.0001$ vs. Surrogate.
